# The impact of the Russian-Ukrainian war on the mental health of Italian people after 2 years of the pandemic: risk and protective factors as moderators

**DOI:** 10.3389/fpsyg.2023.1154502

**Published:** 2023-05-26

**Authors:** Francesca Mottola, Augusto Gnisci, Argyroula Kalaitzaki, Mona Vintilă, Ida Sergi

**Affiliations:** ^1^Department of Psychology, University of Campania “Luigi Vanvitelli”, Caserta, Italy; ^2^Department of Social Work, Hellenic Mediterranean University, Heraklion, Crete, Greece; ^3^Department of Psychology, West University of Timișoara, Timisoara, Romania

**Keywords:** Russian-Ukrainian war, mental health, psychological well-being, moderators, Italian citizens

## Abstract

**Objective:**

This contribution aimed at investigating the moderating role of risk (e.g., the negative influence of COVID-19 on mental health) and protective (e.g., post-traumatic growth) factors on the relationship between the concern for war and stress and anxiety/depression levels among Italian people.

**Methods:**

A questionnaire that included sociodemographic characteristics, the Perceived Stress Scale (PSS-4), the Patient Health Questionnaire (PHQ-4), the Brief Resilience Scale (BRS), the Post-Traumatic Growth Inventory (PTGI), and questions formulated *ad hoc* about concern for war was administered online. A sample of 755 participants (65.4% females, mean age = 32.39, SD = 12.64, range = 18–75) was recruited by convenience and snowball sampling. The researchers spread the link to the questionnaire to their acquaintances asking them to fill it out and to recruit other people.

**Results:**

Results showed that concern for war significantly augmented the levels of stress and anxiety/depression in Italian people. Being a healthcare professional or having a chronic illness negatively moderated the effect of concern for war on stress and anxiety/depression. Instead, the negative influence of COVID-19 on mental health positively moderated the effect of concern for war on stress. Moreover, the overall positive changes after trauma and four of its five scales (i.e., Relating to Others, New Possibilities, Personal Strength, and Spiritual Change), negatively moderated the effect of concern for war on anxiety/depression.

**Conclusions:**

In conclusion, concern about the Russian-Ukrainian war affects the mental health of the Italian population even if they are not directly involved in the conflict.

## 1. Introduction

In 2020, the world was attacked by an insidious virus that changed our daily routines and everything we took for granted until then. We entered the new reality of COVID-19 pandemic rules aiming to contain the pandemic’s deadly effects. Work, education, and social interactions changed in ways that had a significant psychological impact on individuals (Talevi et al., 2020). Even though we are still grappling with the unprecedented scale of disruption the pandemic caused in our lives, we are slowly returning to a less isolated lifestyle. In the midst of this global recovery, since 24 February 2022, the world is experiencing a shocking new reality: the Russian-Ukrainian war. The beginning of this war has dimmed prospects of a post-pandemic economic recovery, triggering a humanitarian crisis throughout Europe as food and commodity prices rose (Orhan, 2022; The Lancet Regional, 2022).

These two crises have also been considered able to compromise physical and mental health globally (Kalaitzaki et al., 2022b), and the relationship between lifetime trauma and vulnerability to the development of mental disorders has been extensively investigated (e.g., Castro-Vale et al., 2020; Silovsky et al., 2022). Together with the COVID-19 pandemic, the war has generated stressors and increased anxiety in different areas of the world (Surzykiewicz et al., 2022).

Much research has been undertaken in the past on the negative effects of wars, terrorist acts, and natural disasters on mental health. Studies in countries that have experienced war and/or armed conflict have shown significant deterioration in mental health among populations directly involved (Bogic et al., 2015; Borho et al., 2020). Recent research has investigated the effects of war on the mental health of citizens directly involved in the Russian-Ukrainian war (Kurapov et al., 2022, 2023; Xu et al., 2023). The results confirm the negative effects of war-related to mental health, fear, substance use, stress, loneliness, burnout, and other related conditions. War-affected populations are at increased risk for mental health problems including PTSD, anxiety and depression (Morina et al., 2018). However, images and information on war posted on social media can also have a negative impact on mental health outside Ukraine. The fear and uncertainty created by the war can have lasting effects on the mental health of Ukrainians and people from other parts of the world, even if they are not directly involved (e.g., Gottschick et al., 2023). The war in Ukraine is the first war in history to be covered almost continuously by the media, and its dramatic scenes and images can be viewed by anyone with access to the Internet or television. Consequently, the psychological negative effects of the war can be felt by the citizens of other countries, even if the effects pale in comparison to those experienced by the citizens of Ukraine (Chudzicka-Czupała et al., 2022). Results have indicated the occurrence of anxiety disorders, acute stress reactions, depressive episodes, cognitive disorders, personality changes, or post-traumatic stress disorder (PTSD) among not only combatants, veterans, and refugees but also among broader groups influenced by mass media coverage of war (Lopez-Ibor et al., 2005; Calderoni et al., 2006; Wahlstrom et al., 2008; Iversen and Greenberg, 2009; Vermetten et al., 2014; Bisson et al., 2015; Johnson et al., 2021). A study on Israeli adults, during the 2014 Gaza war, showed that the frequency of news consumption was associated with anxiety, hyperarousal, and sleeping disorders (Bodas et al., 2015). The negative psychological effects of continued exposure to information about war have contributed to an overall increase in psychopathology, mental health problems, psychosocial dysfunction, self-destruction, and other mental disorders that pose a disease burden for the entire society (Rozanov et al., 2019). Recently, Hoyt et al. (2022) revealed that frequency of exposure to news of traumatic events, such as the COVID-19 pandemic, was a predictor for greater anxiety and depression among United States adults.

Some studies (Brooks et al., 2020; Rubin and Wessely, 2020) have highlighted how the isolation measures, quarantine, and social distancing of the pandemic have affected habitual activities and routines, and this has brought about an increase in loneliness, anxiety, depression, insomnia, harmful use of alcohol and drugs, and self-injurious or suicidal behavior (World Health Organization, 2020). Social isolation as a strategy to contain the spread of COVID-19, was experienced as a traumatic event by young Italian people with pre-existing psychological problems and dysfunctional thinking styles (Giusti et al., 2020). Therefore, undoubtedly, the COVID-19 pandemic had a significant impact on mental health (Cénat et al., 2020; Usher et al., 2020).

Kalaitzaki and Tamiolaki (2022) and Kalaitzaki et al. (2022b) hypothesized that the combined effects of COVID-19 and the war in Ukraine would likely put the mental health of women, the elderly, people with disabilities, and healthcare professionals at serious risk. Indeed, previous studies have found that females, during crisis situations, exhibited more posttraumatic stress disorder (PTSD) or secondary traumatic stress (STS; Kalaitzaki, 2021) and were more prone to anxiety (Wang et al., 2020) than men. Other studies (e.g., Gorrochategi et al., 2020) showed that individuals with a chronic disease appear to experience more symptoms of stress and anxiety than those who do not have a chronic disease during emergency situations, such as COVID-19 pandemic. Moreover, some evidence (e.g., Sergeant et al., 2020) suggests that people with pre-existing mental disorders are more susceptible to the effects of major life stress, such as epidemics and wars. Finally, other scientific evidence has showed that healthcare workers are prone to suffering psychological disorders such as stress, anxiety, and depression due to the specifics of their daily work (Garcia et al., 2022), especially during times of great tribulation. However, compared to non-frontline healthcare workers, frontline healthcare workers were more likely to report anxiety and stress symptoms (Alshekaili et al., 2020).

Italy is not a country directly involved in the ongoing war, but the war has also affected the Italian population because they fear that hostilities could spread beyond Ukraine’s borders. Furthermore, Italy is among the countries with the biggest Ukrainian diaspora. Indeed, over 100,000 Ukrainian sought shelter in Italy after the war started (Mancino, 2022) leading to higher risk of more intensive war-related exposure in the local population as well as a media bombardment process with dire scenes. Long-term or repeated exposure to perceived helplessness is a risk factor for depression, especially when the psychological spectrum of COVID-19 is still present (Jawaid et al., 2022).

Although there is evidence that traumatic events have many negative physical and psychological consequences, many studies examined the importance of personal and social resources (protective factors) that can positively moderate the responses to traumatic events. Researchers have identified multiple risk and protective factors that can impact mental health outcomes during a traumatic experience. Among the identified protective factors there are social support, higher post-traumatic growth (PTG) levels and resilience. Bottomley et al. (2017) showed that social support is central to reducing distress and improving the ability to adapt to traumatic events. Some studies (e.g., Shavitt et al., 2016) have shown that social support is negatively correlated with levels of stressful life events and positively correlated with mental health across cultures. These results were consistent with previous research that has emphasized the importance of social support in decreasing the risk of PTSD (Paoletti et al., 2023). Other studies (Luchetti et al., 2020; Laconi et al., 2021) have also found that being unmarried, lack of social support and loneliness bear a significant mental health toll. These findings were supported by Kalaitzaki (2021) who found that people living with a partner reported less posttraumatic stress disorder/secondary traumatic stress (PTSD/STS), more posttraumatic growth, more frequent use of adaptive coping strategies than those living alone. Some researchers believe that victims are always worried about the negative consequences of stressful events, fearing that the situation will worsen and that their lives will get out of control (Shigemoto et al., 2017). Inquiring about the negative psychological effects of victims’ trauma may prompt the victims to recall the trauma, and this may exacerbate their distress. Instead, focusing on positive psychological changes increases resilience to trauma by strengthening victims’ positivity and reminding them that the trauma did not destroy their entire lives and that the stressful event brought about positive changes (Meyerson et al., 2011).

When faced with a life-threatening event, people tend to reevaluate their goals and priorities, feel more socially connected, and express a greater appreciation of life (Aflakseir et al., 2016). Tedeschi and Calhoun (1996) identified this positive psychological change, which creates a new perception of life after a challenging event, as post-traumatic growth (PTG). PTG can be observed in five domains: valuing interpersonal relationships, greater spiritual gains, greater appreciation of life, discovering new possibilities, and personal strength (Tedeschi and Calhoun, 1996). PTG may coexist with post-traumatic stress symptoms (Kalaitzaki et al., 2022a; Kalaitzaki et al., n.d.), and it may also have a buffering effect on the negative effects of pain and depression (Silva et al., 2012; Wang et al., 2017). Sawyer et al. (2010) also found that PTG was positively associated with positive mental health and subjective physical health but negatively associated with negative mental health. Most studies have found that higher PTG levels are associated with fewer depressive symptoms (Shand et al., 2015). However, some authors (Bianchini et al., 2017) found that personal PTG was predicted by moderate levels of depression in a sample of college students, showing that moderate depressive levels and the related distress could promote the drive to overcome the psychological consequences of the traumatic event. Kalaitzaki et al. (2022c) found emotional social support during the first lockdown and instrumental social support during the second lockdown to predict PTG. While posttraumatic growth is “positive change that an individual experiences as a result of the struggle with a traumatic event” (Calhoun and Tedeschi, 2000, p. 135), resilience is often thought of as the ability to continue living a purposeful life after experiencing hardship or adversity (Thabet, 2017). In contrast to resilience, in which the individual returns to baseline functioning following a highly stressful or traumatic experience, posttraumatic growth is characterized by post-event adaptation that exceeds pre-event levels. Anjum et al. (2023) highlighted that exposure to war-related violence was associated with psychiatric symptoms, while resilience function of character was negatively associated with psychiatric symptoms. However, resilience has been found to correlate moderately with well-being, and higher levels of resilience have been found to be associated with lower levels of reported distress (Kimhi et al., 2020), anxiety, and depression (Barzilay et al., 2020). Resilient individuals are more likely to be proactive in seeking social support and confident in resolving negative events, which has a positive impact on mental health development (Ye et al., 2020). Resilience can transform negative thoughts and feelings into more positive views (Anjum et al., 2023). In a recent study (Xu et al., 2022) a mediated regulation model examined the effects of intrusive rumination on the creativity of college students during the COVID-19 pandemic, as well as the mediating effect of post-traumatic growth and the moderating role of psychological resilience. The results showed that intrusive rumination affected creativity directly and also indirectly through post-traumatic growth. The psychological resilience played a moderating role between intrusive rumination and creativity.

The general aim of this study was to investigate whether and how the war in Ukraine has affected the mental health and well-being of Italian citizens, in order to understand and discover connections and relations among protective factors and stressors. Indeed, we focused on both the negative psychological impact (i.e., anxiety, depression, stress) and the potential positive changes experienced after a traumatic event (i.e., posttraumatic growth in the aspects of relating to others, new possibilities, personal strength, spiritual change, and appreciation of life). In particular, we hypothesized that concern for war would have:

1. A greater negative impact on stress and anxiety/depression levels depending on demographics such as gender (females), age (older), and living conditions (alone), having children (no), those suffering a psychological disorder, chronic illness or underlying diseases;
2. A less negative impact on stress and anxiety/depression levels in resilient people, and in healthcare providers;
3. A greater negative impact on stress and anxiety/depression levels in people who experience the effects of traumatic events (i.e., those informing most frequently themselves about the war, those that COVID-19 has already had a negative impact on their mental health, and those who have less PTG after a traumatic event).

## 2. Materials and methods

### 2.1. Participants

The study involved 755 participants, 491 women (65%) and 260 males (34.4%; 4 missing values), aged between 18 and 75 years old (*M_age_* = 32.39, SD = 12.64). Most of them were married or in a relationship (53.6%), cohabiting (86.9%), had no children (75.8%) and declared not to suffer from any chronic illness/underlying diseases (83.6%) or psychological disorders (87.6%). The majority of the participants had obtained a secondary high school diploma (48.9%), were students (30.6%), were living with their family (68.6%) in urban areas (58.1%) and stated that they used Internet as a reference source for their information about war (54.2%). Approximately one third of the sample were healthcare professionals (33.3%) such as physicians (7.5%), nurses (6.0%), psychologists (1.9%), social workers (0.8%) and para-clinicians (6.1%).

### 2.2. Data collection and procedure

This cross-sectional study was conducted as part of an international project, “The impact of war in Ukraine on mental health”, which aimed to investigate how the war in Ukraine affects the psychological well-being and mental health of people globally. We began the data collection shortly before the election of a new Italian government in 2022. During this period, Italian citizens lived in a context of general social unease related to concerns about the fate of own country from a political point of view, price increases following Russia’s invasion of Ukraine, and the COVID-19 pandemic. Despite a slow global recovery, the human, social, and economic effects of COVID-19 were still highly significant. On top of all this, there was also a concern, exacerbated in that period by the media, of not having enough commodities, such as wheat and gas, which Italy imports from the two countries involved in the conflict. Additionally, we do not underestimate the continuous media exposure of Italian citizens to dramatic scenes and images of war.

The data collection was collected over a one-month period, specifically from 20 September to 24 October 2022, by an online survey. A convenience and snowball sampling technique were used. For this reason, the researchers disseminated the link to the Google Forms questionnaire to their friends, acquaintances, and extended family members. Either through messaging apps (e.g., WhatsApp) or in person, they sent the link and invited their acquaintances to fill out the questionnaire, asking them in turn to spread the link and invite other people. The researchers were involved in spreading the link as much as possible but in focusing, in particular, on identifying also healthcare professional people among their acquaintances. Participants were told that their participation was voluntary, their answers would be confidential, and they could withdraw from the study at any time without any explanation. By entering the online webpage of the survey, participants confirmed that they had read and understood the information about the study and gave their consent to be involved in the research. The study was conducted in conformity with the Declaration of Helsinki requirements, and it was approved by the Ethical Committee of the Hellenic Mediterranean University (no. 87/17-10-2022), in which the principal investigator of the international study (AK) was affiliated.

### 2.3. Measures

A back-translation procedure (Brislin, 1970) was used for scales not already translated and validated in Italian (i.e., resilience, depression and anxiety, and questions about the impact of the war on mental health).

#### 2.3.1. Demographic information

At the beginning of the survey, participants answered demographic questions to provide basic descriptive information such as gender (0 = Males; 1 = Females), age, marital status, educational level, work and, in addition, if they were or not a health professional (i.e., “Are you a health professional working in a health structure (e.g., a hospital)?” – 0 = No; 1 = Yes, a doctor; 2 = Yes, a nurse; 3 = Yes, a psychologist; 4 = Yes, a social worker; 5 = Yes, other paraclinical staff), health personal status (i.e., “Do you suffer from any chronic illness or underlying diseases?” and “Do you suffer from a psychological disorder?” – 0 = No; 1 = Yes), number of children (0–5), and whether or not they lived with other people (“Who do you live with at home?” – 1 = Alone; 2 = Spouse/Partner; 3 = Family; 4 = Other). For the purposes of our analyses, we recoded answer values of health professional question in this way: 0 = No; 1 = Yes. We did the same for the question about number of children (0 = No children; 1 = With children) and cohabiting question (1 = Alone; 2 = With other people).

#### 2.3.2. Concern for the war in Ukraine

Concern for war in Ukraine is defined as the fear of Italian people that they as Italian citizens could be directly involved in the war in the future and that the war itself could any case negatively affects their economic status and psychological state. It included five questions: “Do you think that at some point – sooner or later – our country will also have a war?”; “How stressed are you in the idea that at some point our country might also have a war?”; “How worried are you about the economic crisis that the war in Ukraine has brought about?”; “How much has the economic crisis caused by the war in Ukraine affected you personally?”; “How much do you think the news about the war in Ukraine affects your psychological state?”. Participants were asked to answer using either 5-point Likert scale (i.e., 0 = *Not at all* to 4 = *A lot* or 0 = *Not at all* to 4 = *More than 7 h a week*) either 7-point Likert scale (i.e., 0 = *Not at all* to 6 = *Too much*). The mean of the above five questions defined the Concern for War variable. On these five questions, we carried out a one-factor Principal Component Analysis (PCA). The one-factor solution accounted for 51.76% of the total variance with the unique eigenvalue >1. The Cronbach’s *α* was 0.76.

#### 2.3.3. Perceived stress

We used the four-item version of the Perceived Stress Scale (PSS-4; Cohen et al., 1983) in its Italian translation and adaptation version (Fossati, 2010) to measure the degree to which people assess situations in their lives as stressful. The four-item version was developed as a subset of the longer 10-item version (Cohen et al., 1983). For each item, the respondents rated how often they experienced stressful situations in the previous month using a 5-point Likert scale ranging from 0 (*Never*) to 4 (*Very often*). Two of the PSS-4 items were reverse scored (“In the last month, how often have you felt confident about your ability to handle your personal problems?” and “In the last month, how often have you felt that things were going your way?”). Higher values on the PSS-4 indicate more stress. In this study, the Cronbach’s *α* for this scale was 0.69.

#### 2.3.4. Depression and anxiety

We used the four-item version of the Patient Health Questionnaire (PHQ-4; Kroenke et al., 2009) to measure core symptoms of depression and anxiety. This short form was derived by combining the two-item measure for depression of the Patient Health Questionnaire (PHQ-2; Kroenke et al., 2003) and the two-item Generalized Anxiety Disorder scale (GAD-2; Spitzer et al., 2006). Participants rated the frequency of a given symptom in the past 2 weeks on a 4-point Likert scale, from 0 (*Not at all*) to 3 (*Nearly every day*). Sample items from the scale are “Little interest or pleasure in doing things” and “Feeling down, depressed, or hopeless.” The total PHQ-4 score was extracted by adding together the scores of each of the four items. In this study, the Cronbach’s *α* for this scale was 0.87.

#### 2.3.5. Frequency and sources of war’s information

Two questions were used to assess the frequency with which people inform themselves about war (“How often are you informed about the war in Ukraine?”) and the sources of war’s information (“Where do you get your information from?”). Participants were asked to answer using a 5-point Likert scale, from 1 (*Not at all*) to 5 (*More than 7 h a week*) for the first question; and choosing from the following alternatives for the second question: *TV, Internet, Newspapers, Friends/Acquaintances, Other*. With regard to the first question for the purposes of the analyses, we recoded answer values in this way: 1 = Not at all; 2 = 1–2 h a week; 3 = More than 2 h a week. Instead, for the second question, among the alternatives, we considered only two sources: TV and Internet (1 = TV; 2 = Internet).

#### 2.3.6. Impact of COVID-19 on mental health

One question was developed to assess the impact of COVID-19 pandemic on mental health (“How negatively has COVID-19 affected your mental health overall?”). Participants were asked to answer using a 7-point Likert scale ranging from 0 (*Not at all*) to 6 (*Too much*).

#### 2.3.7. Resilience

The Brief Resilience Scale (BRS; Smith et al., 2008) was used to measure resilience, defined as the ability to bounce back or recover from stress. The scale consists of six statements. The participants rated each of them on a 5-point Likert scale, from 1 (*strongly disagree*) to 5 (*strongly agree*). Sample items from the scale are “It does not take me long to recover from a stressful event” and “It is hard for me to snap back when something bad happens”. Three of the BRS items (“I have a hard time making it through stressful events,” “It is hard for me to snap back when something bad happens”, and “I tend to take a long time to get over setbacks in my life”) were reverse scored. In this study, the Cronbach’s *α* for this scale was 0.83.

#### 2.3.8. Positive changes after traumatic events

The Post-Traumatic Growth Inventory (PTGI; Tedeschi and Calhoun, 1996) in its Italian version (Prati and Pietrantoni, 2014) was used to assess the positive changes experienced after extremely stressful and potentially traumatic events. The scale consists of 21 items organized into five factors: Relating to Others (seven items—for example, “I have more compassion for others”), New Possibilities (five items—for example, “I am able to do better things with my life”), Personal Strength (four items—for example, “I discovered that I’m stronger than I thought I was”), Spiritual Change (two items—for example, “I have a better understanding of spiritual matters”), and Appreciation of Life (three items—for example, “I have a greater appreciation for the value of my own life”). The items are rated using a 6-point Likert scale with values ranging from 0 (*I did not experience this change as a result of my crisis*) to 5 (*I experienced this change to a very great degree as a result of my crisis*). In this study, the Cronbach’s *α* for this scale was 0.96.

### 2.4. Statistical analysis

Descriptive statistics and correlation analysis between key variables were carried out using SPSS 26.0. To control the familywise type I error in correlations, we used the Bonferroni correction. The Hayes (2022) PROCESS macro (model 1) for SPSS and bootstrap procedures (*N* = 5,000) were adopted to examine the moderation models, with Concern for the war as predictor, Stress and Depression-Anxiety as outcome variables, and the following 17 variables as moderators: Sex, Age, Resilience, Healthcare profession, Chronic illness, Number of children, Cohabiting, Psychological disorder, Frequency of war news, Sources of war’s information, COVID-19, Five Aspects of Posttraumatic Growth (Relating to Others, New Possibilities, Personal Strength, Spiritual Change, and Appreciation of Life), and Posttraumatic Growth total score.

## 3. Results

### 3.1. Descriptive statistics and correlation analyses

The distribution of background variables (e.g., gender, age, marital status, etc.) that we will use as moderators in the subsequent analyses has been presented in the Participants section. The descriptive statistics (mean and standard deviation) of all the other variables involved in the study as predictor, outcome variables and moderators are provided in Table 1.

**Table 1:** Descriptive statistics and correlations matrix among predictor, outcome variables and some moderators.

Variables	*M*	*SD*	1	2	3	4	5	6	7	8	9	10	11
1. Concern for war (0–4)	2.17	0.76	1										
2. Stress (0–16)	7.37	3.25	0.28^*^	1									
3. Anxiety/Depression (0–12)	4.89	3.21	0.31^*^	0.66^*^	1								
4. Frequency of war news (1–3)	2.07	1.05	0.15^*^	−0.10	−0.11	1							
5. COVID-19 (0–6)	3.63	1.75	0.42^*^	0.38^*^	0.47^*^	−0.04	1						
6. Resilience (1–5)	3.11	0.84	−0.29^*^	−0.57^*^	−0.51^*^	0.10	−0.41^*^	1					
7. PTG—Relating to Others (0–35)	15.88	8.87	0.06	−0.13	−0.07	0.04	0.09	0.03	1				
8. PTG—New Possibilities (0–25)	12.27	6.44	0.01	−0.16^*^	−0.08	0.02	0.03	0.08	0.79^*^	1			
9. PTG—Personal Strength (0–20)	10.47	5.29	−0.07	−0.24^*^	−0.13^*^	0.02	−0.03	0.20^*^	0.71^*^	0.86^*^	1		
10. PTG—Appreciation of Life (0–15)	8.35	3.95	0.10	−0.10	−0.04	−0.01	0.10	0.05	0.67^*^	0.79^*^	0.76^*^	1	
11. PTG—Spiritual Change (0–10)	3.30	2.78	0.08	−0.06	−0.03	0.10	0.09	0.02	0.56^*^	0.54^*^	0.50^*^	0.48^*^	1
12. PTG—Total Score (0–105)	50.08	24.23	0.03	−0.16^*^	−0.08	0.03	0.06	0.08	0.91^*^	0.94^*^	0.90^*^	0.85^*^	0.66^*^

* Bonferroni correction (*p* < 0.00076).

Table 1 shows also the correlation matrix between those variables. Out of 66 correlations, 31 were significant with Bonferroni correction (*p* < 0.00076). The first group of strongest significant correlations (0.48 < r < 0.94) was between the subscales of the Posttraumatic Growth. The second group (0.28 < r < 0.66) was between the following variables: Concern for war, Stress, Anxiety/Depression, COVID-19, and Resilience. All these last variables correlated positively one each other, apart from Resilience that had a negative correlation with all the others.

We also report the threshold values for the clinical scales used in our research. As far the Stress (PSS-4; Cohen et al., 1983), the 65.7% of our sample obtained a score ranging from 0 to 8 and the 32.7% obtained a score ranging from 9 to 16. According to the threshold values of the Anxiety/Depression scale (PHQ-4; Kroenke et al., 2009), in our sample, the 22.6% fell into the “Normal” category of psychological distress, the 39.7% into “Mild”, the 20.9% into “Moderate” and the 15.5% into “Severe” category. Finally, with regard to the Posttraumatic Growth (Tedeschi and Calhoun, 1996), 50.7% of participants obtained a score ranging from 0 to 52 and the 47.3% ranging from 53 to 105.

### 3.2. The effect of concern for war on stress and the role of the moderators

The results of the moderation analysis for Stress are shown in Table 2.

**Table 2:** Effect of concern for war, of the moderators, and of their interaction on stress.

Moderators	Concern for war				Moderator				Interaction			
	b	S.E.	T	*p*	b	S.E.	T	*p*	b	S.E.	T	*p*
Sex (*N* = 740)	1.13	0.16	7.26	<0.001	−0.61	0.25	−2.42	<0.05	−0.37	0.33	−1.13	0.260
Age (*N* = 744)	1.11	0.15	7.54	<0.001	−0.07	0.01	−7.55	<0.001	−0.02	0.01	−1.87	0.062
Resilience (*N* = 741)	0.54	0.14	3.98	<0.001	−2.04	0.12	−16.84	<0.001	−0.27	0.14	−1.90	0.058
Healthcare profession (*N* = 744)	1.15	0.15	7.63	<0.001	−0.74	0.28	−2.69	<0.01	−1.16	0.36	−3.21	<0.01
Chronic illness (*N* = 730)	1.18	0.15	7.83	<0.001	1.03	0.32	3.24	<0.01	1.06	0.39	2.68	<0.01
Number of children (*N* = 744)	1.18	0.15	8.00	<0.001	−1.64	0.26	−6.27	<0.001	−0.44	0.34	−1.29	0.197
Cohabiting (*N* = 718)	1.13	0.16	7.29	<0.001	−0.45	0.40	−1.11	0.268	0.04	0.53	0.07	0.947
Psychological disorder (*N* = 706)	0.98	0.15	6.52	<0.001	2.64	0.37	7.21	<0.001	−0.11	0.47	−0.23	0.817
Frequency of war news (*N* = 741)	1.32	0.15	8.73	<0.001	−0.68	0.16	−4.30	<0.001	0.36	0.21	1.71	0.088
Sources of war’s information (*N* = 670)	1.15	0.16	7.29	<0.001	0.83	0.25	3.36	<0.001	0.06	0.32	0.19	0.850
COVID-19 (*N* = 659)	0.57	0.17	3.30	<0.001	0.63	0.07	8.54	<0.001	0.18	0.08	2.20	<0.05
PTG—Relating to Others (*N* = 730)	1.24	0.15	8.08	<0.001	−0.05	0.01	−4.02	<0.001	−0.01	0.02	−0.30	0.762
PTG—New Possibilities (*N* = 731)	1.20	0.15	7.87	<0.001	−0.08	0.02	−4.70	<0.001	−0.04	0.02	−1.91	0.057
PTG—Personal Strength (*N* = 732)	1.10	0.15	7.36	<0.001	−0.14	0.02	−6.43	<0.001	−0.04	0.03	−1.44	0.151
PTG—Appreciation of Life (*N* = 732)	1.24	0.15	8.10	<0.001	−0.11	0.03	−3.78	<0.001	−0.04	0.04	−1.01	0.314
PTG—Spiritual Change (*N* = 730)	1.24	0.15	8.04	<0.001	−0.10	0.04	−2.28	<0.05	−0.06	0.05	−1.13	0.258
PTG—Total Score (*N* = 734)	1.19	0.15	7.83	<0.001	−0.02	0.01	−4.84	<0.001	−0.01	0.01	−0.88	0.381

As expected, in all analyses, Concern for war significantly augmented the levels of stress in Italian people. Additionally, all the moderators, apart from Cohabiting, had a significant impact on Stress.

As far as the direction of these effects, males rather than females, younger rather than older people, people with no children compared to people with at least one child, and not being a healthcare professional in comparison to healthcare professionals had higher levels of Stress. People also who were less resilient compared to the more resilient, and people with psychological disorders or with chronic diseases compared to people without, had higher levels of Stress. In terms of the trauma variables, the greater the influence of COVID-19 on mental health, the less informed people about war and the more they received their information via the Internet rather than TV, the higher the levels of Stress. The more positive changes occurred after traumatic events (i.e., total and subscale scores on PTGI), the lower the level of Stress.

Only three interaction effects were significant, that is, three variables moderated the effect of Concern for war on Stress: Healthcare profession, Chronic illness and Negative influence of COVID-19. We present below the analysis of the direction of each effect.

The slope for the interaction effect for Concern for war*Healthcare profession on Stress was significant and negative, explaining an additional 1.3% of the variance in Stress levels (ΔR^2^ = 0.013, *p* < 0.01). As expected, simple slopes analysis showed that, for those who are not healthcare providers, the effect of Concern for war on Stress was significant and positive (b = 1.42, s.e. = 0.17, t = 8.26, *p* < 0.001), while for healthcare providers it was no longer significant (b = 0.26, s.e. = 0.32, t = 0.81, *p* = 0.419).

The slope for the interaction effect for Concern for war*Chronic illness or underlying diseases on Stress was significant and positive. It explained an additional 0.9% of the variance (ΔR^2^ = 0.009, *p* < 0.01). Simple slopes analysis showed that, for both those who suffered and those who did not suffer from chronic illness or underlying diseases, the moderation effect was significant and positive. However, in line with our expectation, for those who suffered from chronic illness or underlying diseases, the effect (b = 2.07, s.e. = 0.36, t = 5.80, *p* < 0.001) was higher than for those who did not (b = 1.02, s.e. = 0.17, t = 6.17, *p* < 0.001).

Finally, the slope for the interaction Concern for war*Negative influence of COVID-19 on Stress was significant and positive. The moderation explained an additional 0.6% of the variance in Stress levels (ΔR^2^ = 0.006, *p* < 0.05). Simple slopes showed that, for people whose mental health was severely damaged by the COVID-19 pandemic, the effect of Concern for war on Stress was significant and positive (b = 0.88, s.e. = 0.23, t = 3.82, *p* < 0.001), while for people on whom the pandemic had not had a negative impact, the effect on Stress was not significant (b = 0.26, s.e. = 0.22, t = 1.19, *p* = 0.236).

### 3.3. The effect of concern for war on anxiety/depression and the role of the moderators

The results of the moderation analysis for Anxiety/Depression are shown in Table 3. In all analyses, Concern for war significantly increased Anxiety/Depression. More, out of the 17 moderators, 13 had a main effect on Anxiety/Depression.

**Table 3:** Effect of concern for war, of the moderators, and of their interaction on anxiety/depression.

Moderators	Concern for war				Moderator				Interaction			
	b	S.E.	T	*p*	b	S.E.	T	*p*	b	S.E.	T	*p*
Sex (*N* = 742)	1.27	0.15	8.13	<0.001	−0.86	0.24	−3.54	<0.001	0.34	0.32	1.06	0.290
Age (*N* = 746)	1.23	0.14	8.72	<0.001	−0.07	0.01	−8.64	<0.001	−0.01	0.01	−1.21	0.226
Resilience (*N* = 741)	0.77	0.14	5.62	<0.001	−1.75	0.12	−14.22	<0.001	−0.13	0.14	−0.90	0.368
Healthcare profession (*N* = 746)	1.30	0.15	8.82	<0.001	−0.61	0.27	−2.27	<0.05	−1.06	0.35	−2.98	<0.01
Chronic illness (*N* = 732)	1.33	0.15	9.07	<0.001	0.93	0.31	2.99	<0.01	0.93	0.38	2.42	<0.05
Number of children (*N* = 746)	1.32	0.14	9.21	<0.001	−1.79	0.25	−7.10	<0.001	−0.20	0.33	−0.60	0.550
Cohabiting (*N* = 720)	1.30	0.15	8.61	<0.001	−0.03	0.39	−0.07	0.943	−0.22	0.51	−0.43	0.666
Psychological disorder (*N* = 708)	1.08	0.14	7.55	<0.001	3.17	0.35	9.11	<0.001	−0.09	0.44	−0.19	0.847
Frequency of war news (*N* = 743)	1.45	0.15	9.82	<0.001	−0.78	0.15	−5.06	<0.001	0.34	0.20	1.67	0.096
Sources of war’s information (*N* = 672)	1.28	0.15	8.38	<0.001	0.89	0.24	3.76	<0.001	0.24	0.31	0.77	0.441
COVID-19 (*N* = 660)	0.44	0.16	2.66	<0.01	0.79	0.07	11.29	<0.001	−0.04	0.08	−0.54	0.590
PTG—Relating to Others (*N* = 730)	1.34	0.15	8.89	<0.001	−0.03	0.01	−2.52	<0.05	−0.04	0.02	−2.66	<0.01
PTG—New Possibilities (*N* = 731)	1.33	0.15	8.93	<0.001	−0.05	0.02	−2.66	<0.01	−0.05	0.02	−2.45	<0.05
PTG—Personal Strength (*N* = 732)	1.26	0.15	8.46	<0.001	−0.07	0.02	−3.34	<0.001	−0.06	0.03	−2.33	<0.05
PTG—Appreciation of Life (*N* = 732)	1.35	0.15	8.95	<0.001	−0.06	0.03	−2.04	<0.05	−0.07	0.04	−1.86	0.064
PTG—Spiritual Change (*N* = 730)	1.37	0.15	9.14	<0.001	−0.07	0.04	−1.69	0.092	−0.11	0.05	−2.02	<0.05
PTG—Total Score (*N* = 734)	1.29	0.15	8.61	<0.001	−0.01	0.01	−2.55	<0.05	−0.01	0.01	−2.17	<0.05

As far as the direction of these effects, males rather than females, younger rather than older people, people with no children compared to people with at least one child, and not being a healthcare professional compared to professionals had higher levels of Anxiety/Depression. People less resilient compared to the more resilient, and people with psychological disorders or chronic illness compared to those who had not, had higher levels of Anxiety/Depression. In terms of trauma variables, the greater the influence of COVID-19 on mental health, the less informed people about war and the more they received their information via the Internet rather than TV, the higher the levels of Anxiety/Depression. The more positive changes occurred after traumatic events (i.e., total and subscale scores on PTGI, except for Appreciation of Life subscale), the lower the level of Anxiety/Depression.

Seven significant interaction effects arose between Concern for war and the following moderators: Healthcare profession, Chronic illness, overall Positive changes after trauma and four of its aspects (Relating to Others, New possibilities, Personal Strength, and Spiritual Change). We present below the analysis of the direction of each effect.

The slope of the interaction Concern for war*Healthcare profession on Anxiety/Depression was significant and negative, explaining an additional 1.1% of the variance in Anxiety/Depression levels (ΔR^2^ = 0.011, *p* < 0.01). As expected, simple slopes showed that, for those who were not healthcare providers, the effect of Concern for war on Anxiety/Depression was significant and positive (b = 1.54, s.e. = 0.17, t = 9.22, *p* < 0.001) while for healthcare providers it was no longer significant (b = 0.49, s.e. = 0.31, t = 1.56, *p* = 0.121).

The slope of the interaction effect Concern for war*Chronic illness or underlying diseases was significant and positive, explaining an additional 0.7% of the variance in Anxiety/Depression levels (ΔR^2^ = 0.007, *p* < 0.05). Simple slopes analysis showed that the effect of Concern for the war on Anxiety/Depression was significant and positive for both people who were not chronically ill (b = 1.19, s.e. = 0.16, t = 7.39, *p* < 0.001) and those who were chronically ill (b = 2.12, s.e. = 0.35, t = 6.09, *p* < 0.001), even if, in the latter case, the effect was much more positive. This outcome is in line with our expectation.

The slope of the interaction effect between Concern for war and the total score of the PTGI on Anxiety/Depression was significant and negative, explaining an additional 0.6% of the variance in Anxiety/Depression levels (ΔR^2^ = 0.006, *p* < 0.05). Simple slopes analysis showed that both in the absence of positive change after a traumatic event (mean = 1SD; b = 1.60, s.e. = 0.20, *t* = 8.10, *p* < 0.001) and in the presence of great positive change experienced after a traumatic event (b = 0.98, s.e. = 0.22, *t* = 4.53, *p* < 0.001), Concern for war increased Anxiety/Depression, even if, in line with our expectations, in people who experienced positive change after a traumatic event, the war affected Anxiety/Depression less than in people who did not experience positive changes. The specific interaction effects between Concern for War and the four subscales of PTGI followed the same pattern. Simple slopes analysis showed that in people who experienced positive changes after a traumatic event in Relating with Others (ΔR^2^ = 0.009, *p* < 0.01; b = 0.96, s.e. = 0.22, *t* = 4.35, *p* < 0.001), Having New Possibilities (ΔR^2^ = 0.007, *p* < 0.05; b = 0.98, s.e. = 0.21, *t* = 4.63, *p* < 0.001), Personal Strength (ΔR^2^ = 0.007, *p* < 0.05; b = 0.94, s.e. = 0.21, *t* = 4.50, *p* < 0.001), and Spiritual Change (ΔR^2^ = 0.005, *p* < 0.05; b = 1.07, s.e. = 0.21, *t* = 5.09, *p* < 0.001), the effect of Concern for war on Anxiety/Depression was positive, as it was for people who did not have that positive experience (respectively, b = 1.72, s.e. = 0.20, *t* = 8.81, *p* < 0.001; b = 1.68, s.e. = 0.20, *t* = 8.36, *p* < 0.001; b = 1.58, s.e. = 0.20, *t* = 7.99, *p* < 0.001; b = 1.67, s.e. = 0.21, *t* = 7.99, *p* < 0.001). However, for the people who experienced positive changes, the effect was weaker than for the people who did not have that positive experience, and this result aligned with our expectation.

## 4. Discussion

The general aim of this study was to investigate whether and how the war in Ukraine affected the mental health and psychological well-being of Italian citizens, considering the moderating role of some risk (e.g., the negative impact of COVID-19 on mental health) and protective factors (e.g., resilience). To answer this question, we first provide a discussion of the main general results of the present research and then we will pass to discuss in detail each point.

First, the present research demonstrated that the occurrence of a war, the fear for its consequences and the concern of being involved may have a negative effect on levels of stress and anxiety/depression also on people not directly involved in the war, as the Italian citizens. Second, almost all the variables we considered had a direct impact on stress and anxiety/depression on these people during a time of crisis and that many of them may act as risk or protective factors for improving or aggravate their mental health. Indeed, our research demonstrates that many background variables (as age, sex, healthcare profession and number of children), pathologic conditions (psychological disorders, chronic illness), individual psychological resources as resilience and abilities to face traumatic events, contextual variables connected to exposure to and source of war news and, finally, the serious problematic situation due to the past pandemic, all of these variables may strongly impact, in different ways, on the levels of stress and anxiety/depression. Third, our research demonstrated that some of these variables may moderate, in a positive or negative way, the effect of concern for war on stress and anxiety/depression.

As far as specific results of our research regard, we have seen that gender and age had a significant, negative main effect on both stress and anxiety/depression levels. Contrary to the results of previous studies (e.g., Mohsen et al., 2021; Riad et al., 2022), which have shown that women, children, and elderly people are more vulnerable in crisis situations, we did not find age and gender to moderate the relationship between concern for war and stress and anxiety/depression. Therefore, the hypothesis that women’s and older people’s mental health is more at risk in crisis situations, such as a war, was not confirmed. With regard to age, this result can be due that, the elderly, during their lifetime, have been exposed to higher number of potential traumatic experiences compared to young people, and this had allowed them to acquire/develop useful skills to successfully deal with similar experiences. Similarly, resilience showed a significant negative main effect on both stress and anxiety/depression levels, in line with the results of previous studies (Barzilay et al., 2020; Kimhi et al., 2020; Maftei et al., 2022; Anjum et al., 2023) which have been found that higher levels of resilience were associated with lower levels of reported distress, anxiety, and depression. But contrary to the assumptions, it did not moderate the relationship between concern for war and stress and anxiety/depression levels. We consider “having children” as a protective factor. Specifically, we start with the assumption that that people with children did not experience loneliness and had a greater social support than those without children. Indeed, as we have seen from previous literature (e.g., Shavitt et al., 2016), both loneliness and lack of social support (Paoletti et al., 2023) have a significant negative impact on mental health. In line with this, we found a negative main effect of the number of children on mental health. But contrary to our assumptions, this variable did not moderate the relationship between concern for war and stress and anxiety/depression levels. Psychological disorder had a main negative effect on stress and anxiety/depression levels, in line with some evidence from previous studies (e.g., Sergeant et al., 2020) which suggested that people with pre-existing mental disorders are more vulnerable to the effects of high life stress. Despite this, contrary to our assumptions, psychological disorder did not moderate the relationship between concern for war and mental health. Similarly, and in line with the hypothesis of Hoyt et al. (2022), the results of this study showed a significant and negative effect of the frequency with which people inform themselves about the war on stress and anxiety/depression levels. But despite this, the frequency of following war news did not moderate the relationship between the concern for war and stress and anxiety/depression levels. Literature has clearly suggested that healthcare workers, due to the specifics of their daily work, tend to suffer from psychological disorders such as stress, anxiety, and depression (Garcia et al., 2022), but it also suggested that such risks were greater for frontline healthcare workers compared to non-frontline healthcare workers (Alshekaili et al., 2020) as in the case of Italian healthcare workers. Moreover, healthcare workers, due to their work, are exposed to multiple problematic medical situations, and in cases like this in which war is not experienced directly, this, according to us, can be a protective factor. In line with our hypothesis and the findings of the previous studies, the results showed that being a healthcare professional (non-frontline) moderated the relationship between concern for war and both stress and anxiety/depression; in particular, the mental health of people who were lay persons was mostly affected by concern for the war in Ukraine. Although the results showed that both suffering and not suffering from chronic illnesses positively affected the relationship between concern for war and mental health, people who suffered from chronic illness or underlying diseases were mostly affected by concern for war. These results are in line with those of previous studies (e.g., Gorrochategi et al., 2020) which have shown that individuals with a chronic disease experience more symptoms of stress and anxiety than those who do not have a chronic disease during crisis situations. Another important aspect not to be underestimated is the negative impact that the COVID-19 pandemic has already had on mental health. Literature has clearly indicated that the COVID-19 pandemic negatively impacted mental health (e.g., Barchielli et al., 2022). The current study revealed that the COVID-19 pandemic had a main positive effect on high levels of stress and anxiety/depression, and that it also had an interaction effect between concern for war and the COVID-19 pandemic on stress levels but not on anxiety/depression levels. To our knowledge, this is the first study that has investigated the role of the PTG factors as moderators in the relationship between a traumatic event and mental health. In line with findings showing that PTG is associated with positive mental health (Sawyer et al., 2010), our study showed that total score and all five PTG subscale scores negatively affected high levels of stress and anxiety/depression. Moreover, PTG total and subscale scores, except for the Appreciation of Life subscale, moderated the relationship between concern for war and anxiety/depression levels, but not for stress levels.

In conclusion, the Russian invasion of Ukraine has had a negative impact on the well-being and mental health of Italian people. Additionally, the study suggests that the invasion affected citizens of countries not directly being involved in the war, causing increased levels of anxiety and depression. However, our research demonstrates that many factors–whether they are sociodemographic, individual, or psychological factors, or whether they are related to exposure to war, the COVID-19 pandemic, or the capability to positively react to traumatic events (i.e., PTG)—have a direct impact on mental health and, in some cases, they reduce or increase the risk with which the war and the concern about it determines mental health problems.

The results of this study might provide guidelines to develop clinical interventions aimed at coping with difficult living circumstances. Specifically, psychotherapy should focus on three situations: the refugees themselves, citizens of countries that are not directly involved in the war, such as Italians in our study, and professional caretakers. Until now several psychotherapeutic models have been applied and assessed related to PTSD of refugees and in war affected regions: Trauma-Focused Cognitive-Behavioral Therapy (Anjum et al., 2023), Emotional Schema Therapy, Narrative Exposure Therapy, Integrative Gestalt Derived Intervention (Jacob et al., 2014; Kira and Tummmala-Narra, 2014; Butollo et al., 2016; Rajeh et al., 2017). Each of them proved its efficiency and that the effects were maintained at re-evaluation after 6/12 month. They helped decrease anxiety, depression and controlling symptoms of PTSD. The existing studies focus on refugees and their unique challenges, not on citizens of other countries and how they experience this specific situation, given the large number of refugees, the huge media exposure, social-financial burden etc. In the future, based on our study, specific interventions should be designed for the three target groups mentioned above with the purpose of focusing on resilience and posttraumatic growth.

This study has great significance in considering the impact of war in Ukraine on a wide and large sample of Italian citizens’ mental health. Despite this, the study had some limitations. First, the design was cross-sectional, which is not very suitable for assessing causality since the temporality of association cannot be checked. Second, the sample had a higher percentage of females than males and of younger people (*M_age_ =* 32.39) than older people, thus the generalizability of our results can be limited. Third, the participants were recruited, using a convenience and snowball sampling procedures, mainly from central and southern Italy, consequently, the generalizability of our findings can be limited compared to a national sample. Finally, for the measure of some psychological aspects (such as, the impact of COVID-19 pandemic on mental health) we used only one item: future studies should use scales with more items to adequately represent the construct.

## Data availability statement

The raw data supporting the conclusions of this article will be made available by the authors, without undue reservation.

## Ethics statement

The studies involving human participants were reviewed and approved by Ethical Committee of Hellenic Mediterranean University (no. 87/17-10-2022). The participants provided their electronic informed consent to participate in this study.

## Author contributions

FM translated and adapted the Italian instruments not previously validated, contributed to data collection, performed the statistical analysis, wrote part of the manuscript, and critically reviewed it. AG formulated the research questions/hypotheses, supervised the data collection and analysis, interpreted the results, wrote part of the manuscript, and critically reviewed it. AK and MV designed the study, prepared the questionnaire, supervised the data collection, and critically reviewed the manuscript. IS prepared and adapted the Italian measure instruments not previously validated, supervised the data collection, and wrote part of the manuscript. All authors contributed to the article and approved the submitted version.

## Funding

The research leading to these results has received funding from the program V:ALERE 2019 of the University of Campania “Luigi Vanvitelli” (D.R. 906 del 4/10/2019, prot. n. 157264, 17/10/2019).

## Conflict of interest

The authors declare that the research was conducted in the absence of any commercial or financial relationships that could be construed as a potential conflict of interest.

## Publisher’s note

All claims expressed in this article are solely those of the authors and do not necessarily represent those of their affiliated organizations, or those of the publisher, the editors and the reviewers. Any product that may be evaluated in this article, or claim that may be made by its manufacturer, is not guaranteed or endorsed by the publisher.
